# Presence of Circulatory Autoantibodies Against ROS-Modified Histone H1 Protein in Lymphoma Patients

**DOI:** 10.3389/fgene.2022.909903

**Published:** 2022-05-25

**Authors:** Naif K. Binsaleh, Reem Eltayeb, Husam Qanash, Mohammad Azhar Aziz, Raid Albaradie, Mohd Wajid Ali Khan

**Affiliations:** ^1^ Department of Medical Laboratory Science, College of Applied Medical Sciences, University of Ha’il, Ha’il, Saudi Arabia; ^2^ Molecular Diagnostics and Personalized Therapeutics Unit, University of Ha’il, Ha’il, Saudi Arabia; ^3^ Interdisciplinary Nanotechnology Centre, Aligarh Muslim University, Aligarh, India; ^4^ Applied Medical Sciences College, Majmaah University, Al Majma’ah, Saudi Arabia; ^5^ Department of Chemistry, College of Sciences, University of Ha’il, Ha’il, Saudi Arabia

**Keywords:** lymphoma, Hodgkin lymphoma, non-Hodgkin lymphoma, autoantibodies, reactive oxygen species, oxidative stress, histone H1

## Abstract

Lymphoma is a chronic inflammatory disease in which the immune system is highly affected. Increased oxidative stress is one of the common conditions of cancer and affects macromolecules. Histone modifications affect the chromatin structure and functions. In this study, histone H1 (His-H1) protein was modified by reactive oxygen species (ROS), and structural and chemical changes were studied. Hodgkin lymphoma (HL) and non-Hodgkin lymphoma (NHL) patients were selected, and oxidative stress markers, inflammatory cytokines, and serum autoantibodies were analyzed using biochemical and immunological assays. Furthermore, the formation of antigen-antibody immune complexes was assessed by the Langmuir plot. ROS-modified His-H1 (ROS-His-H1) showed substantial structural perturbation in protein (UV-hyperchromicity and increased intrinsic fluorescence) compared to the native His-H1 protein. A possible explanation for the changes is suggested by the exposure of the aromatic chromophore to the solvent. In-depth structural analysis by circular dichroism (CD) exhibited major changes in α-helix (−21.43%) and turns (+33%), reflecting changes in the secondary structure of histone H1 protein after ROS exposure. ELISA and competitive ELISA findings revealed high recognitions of serum autoantibodies to ROS-His-H1 from NHL, followed by HL subjects. Healthy controls showed negligible binding. Non-modified His-H1 did not show any binding with serum samples from either cohort. High apparent association constants (ACCs) were calculated for ROS-His-H1 using purified IgGs from NHL (1.46 × 10^–6^ M) compared to HL (1.33 × 10^–6^ M) patients. Non-modified His-H1 exhibited a hundred times less ACC for NHL (2.38 × 10^–8^ M) and HL (2.46 × 10^–8^ M) patients. Thus, ROS modifications of histone H1 cause structural changes and expose cryptic neo-epitopes on the protein against which autoantibodies were generated. These perturbations might affect the histone DNA interaction dynamics and potentially be correlated with gene dysregulation. These subtle molecular changes with an immune imbalance might further aggravate the disease.

## Introduction

Lymphoma is a broad term for cancers that arise from the clonal proliferation of lymphocytes. Although they are generally categorized into Hodgkin lymphoma (HL) and non-Hodgkin lymphoma (NHL), the term lymphoma encompasses over 50 different subtypes ranging from low-grade, slow-growing cancers to high-grade, aggressive neoplasms (Mugnaini and Ghosh, 2016). The heterogeneous nature of lymphoma, in terms of etiology, pathogenesis, and patient demographic, is also reflected in patient prognosis; some types of NHL such as mantle cell lymphoma have 5-year survival rates of 40%, while others such as marginal zone lymphoma have 5-year survival rates of approximately 90%. (Jamil and Mukkamalla, 2022).

An association between the degree of oxidative stress in lymphoma patients and a poor prognosis has been reported (Nakazato et al., 2014). In fact, so strong is this association that markers of oxidative stress have been demonstrated to be an independent predictive factor for survival in patients with the NHL subtype diffuse large B-cell lymphoma (DLBCL) (Nakazato et al., 2014). Oxidative stress refers to a state in which there is an imbalance between the production and degradation of ROS (Pizzino et al., 2017). ROS are produced as by-products of cellular metabolism in all cells, and due to the presence of a single unpaired electron, they are highly reactive and must, therefore, be neutralized. Indeed, it has been proven beyond doubt that ROS can damage DNA and other biomolecules (Juan et al., 2021). Production and degradation of ROS within normal cells are, therefore, finely controlled by redox state-regulating enzymes. Excess levels of ROS in normal cells result in apoptosis, yet cancer cells appear to be able to tolerate higher excess levels and hence can undergo further ROS-induced damage which can drive tumorigenesis (Benassi et al., 2006).

The source of elevated ROS in cancer patients is still unclear. It is well established that cancer cells themselves produce higher levels of ROS mainly due to the increased metabolic activity and mitochondrial dysfunction (Storz, 2005). However, ROS production is also associated with chronic inflammation, and the link between inflammation and cancer has long been established (Vendramini-Costa and Carvalho, 2012; Greten and Grivennikov, 2019; Wu et al., 2021). It has been demonstrated that during inflammation, in which there are high levels of local and systemic pro-inflammatory cytokines, the increased activity of phagocytes results in increased ROS generation, and this itself can promote tumorigenesis (Zhang and An, 2007; Dupré-Crochet et al., 2013; Snezhkina et al., 2019).

Histones are cationic proteins that associate with DNA and play an important role in stabilizing the chromatin structure. DNA is packed into chromatin by histones to form a tightly coiled structure in the nucleus. Histones undergo several post-translational (epigenetic) modifications such as methylation, acetylation, phosphorylation, ubiquitination, and ADP ribosylation (Kim et al., 2015). The remodeling of chromatin by histone modification plays a key role in the regulation of cellular processes such as DNA repair, gene transcription, cell differentiation, and cell proliferation (Grewal and Jia, 2007; Feng et al., 2010; Kanherkar et al., 2014). Irregular patterns of histone modifications have been reported in a wide range of human cancers (Audia and Campbell, 2016) and are associated with the deregulation of chromatin-based processes. It has been proposed that ROS may be directly responsible for histone modification and might result in oncogenic transformation (Afanas’ev, 2013). Furthermore, ROS-induced histone modifications could be used as potential biomarkers of cancer progression and prognosis. Autoantibodies against modified histones are present in a number of autoimmune diseases such as systemic lupus erythematosus (SLE) and rheumatoid arthritis (RA) (Xiaoyu and Yumin 2019). Interestingly, patients with autoimmune conditions develop neoplastic diseases, especially lymphoma, more frequently than the general population (Swissa et al., 1992).

We sought to determine the structural modifications to histone H1 (His-H1) that result from ROS and whether autoantibodies present in lymphoma patients are specific for this modified histone. His-H1 was modified *in vitro* by ROS and was then used as an antigen in direct binding ELISA and inhibition ELISA to study the specificity of the autoantibodies in the serum from HL and NHL patients. The antigen-antibody affinity was assessed by the Langmuir plot. Furthermore, the pro-inflammatory cytokines interferon-γ (IFN-γ), tumor necrosis factor-α (TNF-α), and interleukin-6 (IL-6) in HL and NHL patients were studied and compared to those of healthy controls to further understand the role of cytokines in the pathogenesis of lymphoma.

## Materials and Methods

### ROS Modification of Histone H1

The H1 histone protein was modified by the hydroxyl radical (˙OH). An aqueous solution of His-H1 protein (3 mg/ml) in PBS (pH 7.4) was irradiated under ultraviolet (UV) radiation (254 nm) for 30 min at 25°C in the presence of hydrogen peroxide (10 mM). These conditions will generate ˙OH. After the reaction, excess hydrogen peroxide and ˙OH were removed by extensive dialysis against PBS.

### UV Spectrophotometry

Native and ROS-His-H1 proteins were analyzed on a UV-1700 Shimadzu spectrophotometer (Kyoto, Japan) using a range of 250–500 nm wavelength. A quartz cuvette of 1 cm path length was used at 25°C.

### Fluorometry

Native and ROS-His-H1 proteins were analyzed for fluorescence spectra. It was performed using a spectrofluorophotometer (Shimadzu RF-5301-PC, Tokyo, Japan) at 25 ± 0.1°C in a cell of 1-cm path length with a slit width of 3 nm. Intrinsic fluorescence was measured when both samples were excited at the wavelength of 275 nm, and the emission spectra were recorded at 300–400 nm of the wavelength range. The change in the fluorescence intensity due to modification was calculated as follows:

$$\mathrm{Percent}\;\mathrm{change}\;\mathrm{in}\;\mathrm{fluorescence}=\frac{\mathrm{Native}\;\mathrm{His}-H1-\mathrm{ROS}-\mathrm{His}-H1}{\mathrm{Native}\;\mathrm{His}-H1}\times 100.$$

### Circular Dichroism

Secondary structural perturbation was identified by CD as published earlier (Ali Khan et al., 2007). All the protein samples, with or without inhibitors, were run on a Jasco J-810 spectropolarimeter equipped with a temperature-controlled sample cell holder attached to a NESLAB model RYE 110 water bath with an accuracy of ±0.10°C. The range used to analyze CD spectra was between 200–280 nm at 5 mm/millidegree (mdeg) sensitivity. Samples were prepared in PBS, pH 7.4. The Chen and Yang equation was used to estimate the relative percentage of secondary structures (Chen and Yang 1971). Each sample was run three times.

### Serum Sample Collection

Serum samples from patients (HL and NHL) and healthy controls were collected in university clinics after receiving their full written consent. This study has been approved by the institutional ethics committee, and the details are given at the end in the ‘Ethics Statement’ section. Up to 3 ml of serum samples were collected from all subjects. Patients with other comorbidities were excluded from the study. Pregnant women and individuals less than 18 years of age were also excluded from the study. Levels of C-reactive protein (CRP) were measured using the latex agglutination reaction method. All lymphoma patients were using prescribed drugs and therapies (details have not been provided). Demographic details such as age, gender, smoking duration, and disease duration of the subjects were included in the study (Table 1).

**TABLE 1:** Clinical characterizations of lymphoma patients (HL and NHL) and healthy individuals.

Group	Age (years)	Gender (men/women)	Duration of disease	C-reactive protein (mg/L)	IFN-γ (pg/ml)	TNF-α (pg/ml)	IL-6 (pg/ml)	Smoking duration (years ±SD)
HC (*n* = 15)	36 ± 8.3	9/6	—	0.96 ± 0.74	3.2 ± 2.15	0.91 ± 0.18	2.1 ± 1.84	—
HL (*n* = 15)	38 ± 6.2	10/5	7.3 ± 3.1	6.88 ± 4.1***	4.1 ± 2.4*	1.21 ± 0.22**	8.2 ± 6.2***	9.2 ± 4.7
NHL (*n* = 15)	41 ± 5.7	9/6	8.1 ± 3.7	7.34 ± 4.4***	4.4 ± 2.1*	1.43 ± 0.21**	8.7 ± 5.8***	11.3 ± 5.4

All tests for each serum sample were run in triplicate. n represents the number of samples. All values are given as mean ± standard deviation (SD). *p < 0.05, **p < 0.01, and ***p < 0.001.

### Detection of Carbonyl Compounds

The carbonyl content is also considered an oxidative stress for proteins. Both native and ROS-modified histone H1 samples were analyzed for the detection of carbonyl contents attached to the protein molecules using the previously published method (Levine et al., 1990). Quenching studies using mannitol (100 mM), catalase (500 units/ml), DETAPAC (100 mM), and SOD (500 units/ml) were performed for carbonyl compounds.

A similar method was employed to identify the oxidation of protein in cancer patients’ serum samples. Serum samples from healthy individuals were also analyzed for comparative analysis.

### Serum Malondialdehyde Contents

The MDA content is a marker for lipid oxidative stress and was estimated in all serum samples. A commercially available MDA assay kit was used, according to the manufacturer’s instructions (Elabscience, United States). Briefly, plasma/serum samples were used in the assay. Results were measured at 532 nm absorbance on an ELISA reader, and the values were given as nmol/ml. The amount of MDA contents was estimated using the given formula:

$$\mathrm{MDA}=\frac{\Delta \mathrm{A1}}{\Delta \mathrm{A2}}\times c.$$

ΔA_1_; OD of sample—OD of control.

ΔA_2_; OD of standard—OD of blank.

C; concentration of standard (10 nmol/ml).

### Cytokine Estimation

Cytokines IFN-γ, TNF-α, and IL-6 were analyzed in healthy control and patients’ serum samples based on quantitative sandwich immunoassay (R&D System, Minneapolis, MN, United States) with a sensitivity of less than 0.5 pg/ml for all cytokines. All samples were assayed in triplicate.

### Isolation of IgG by Protein A Agarose

IgG from patients’ serum was isolated and purified by using a protein A agarose column (affinity chromatography) (Sigma-Aldrich, United States). Serum samples were mixed with an equal volume of phosphate buffer saline (pH 7.4) and then applied to the protein A agarose column. The column was washed with the same buffer two to three times to remove unbound IgG. The bound immunoglobulins were eluted with NaCl (0.85%) and acetic acid (0.58%) using methods published by Goding with slight modifications (Goding, 1978), and the reaction mixtures were neutralized by adding 1 ml of Tris HCl (1M, pH 8.5). Then, 3 ml fractions were collected, and the reading was observed at 251 and 278 nm to determine the concentration of IgG by taking 1.40 OD_280_ = 1.0 mg/ml.

### Immunological Detection of Antibodies

Direct binding ELISA: Autoantibodies were detected by ELISA with the help of polystyrene microtiter plates using a solid support, as published earlier (Alouffi et al., 2018; Khan et al., 2020). A measure of 100 μL of ROS-His-H1 and His-H1 protein antigens (5 μg/ml) in Tris-buffered saline (TBS) (10 mM Tris and 150 mM NaCl; pH 7.4) were coated on microtiter plates. The reaction mixtures were incubated at 37°C initially for 2 h and then for 24 h at 4°C. Antigen-containing wells were washed three times with the same buffer to remove the unbound antigen. The unoccupied site on the antigen was blocked by 1.5% bovine serum albumin (150 µL) in TBS for 4 h. The plates were washed again, and antibodies pre-diluted with TBS were added to each well (100 µL/well). The plates were incubated for 2 h at 37°C and 24 h at 4°C. Then, plates were washed three times with Tris-buffered saline Tween 20 (TBS-T) (20 mM Tris, 144 mM NaCl, 2.68 mM KCl, pH 7.4, containing 500 µL Tween/L). After that, a suitable IgG alkaline phosphatase conjugate was added to each well. The plates were washed again three times with TBS-T and two times with distilled H_2_O, after incubation for 2 h at 37°C. The reaction was developed by using p-nitrophenyl phosphate as a substrate, and the readings were recorded at 410 nm onto a microplate reader (MR9600-415 Accuris, NJ, United States). Control wells were given the same condition, but the wells were devoid of any antigen. Each sample was run in triplicate, and results were considered as a mean of A_test_─A_control_.

Inhibition ELISA: The specificity of the antibodies was estimated by inhibition ELISA. Different amounts of competitors from 0 to 20 μg/ml were incubated with a constant amount of serum IgG for 2 h and later for 24 h at 4°C. The immune complex, rather than serum/IgG, was coated on the microtiter plates. The remaining procedure followed the same, as indicated in direct binding ELISA. Percentage inhibitions were calculated as given previously (Alouffi et al., 2018; Khan et al., 2020).

### Quantitation of Immune Complex by the Langmuir Plot

An increasing amount of (0–40 μg) of ROS-His-H1 protein was incubated with a constant amount of purified IgG from patients and healthy control subjects at 37°C for 2 h at room temperature. Then, the complexes were transferred to the fridge (4°C) for overnight incubation for further antigen-antibody binding. Immune complexes thus formed, were pelleted, followed by washing twice with PBS, and finally dissolved in 1N sodium chloride (250 μL). Protein concentrations were estimated by using a nanodrop and commercially available BCA protein assay kit (Sigma-Aldrich, United States). Data from the immunoprecipitation reactions were analyzed for the calculations of the apparent association constant (ACC) of the bound antigen and antibody in an equilibrium mixture by the method of Langmuir (1918).

### Statistical Analysis

Significant differences between control values were determined with Student’s *t*-test (SPSS 16.0, Chicago, United States). A *p*-value of <0.05 was indicative of statistical significance.

## Results

### Clinical Characterization of Patients

Clinical characterization of HL and NHL patients was investigated for CRP and cytokine levels (IFN-γ, TNF-α, and IL-6) (Table 1). A significant increase (*p* < 0.01) in the levels of CRP and TNF-α was observed in both categories of cancer patients compared to the healthy individuals. A slight increase in the level of INF-γ was reported in cancer patients compared to the healthy controls.

### Biophysical Characterization of ROS-Modified His-H1 Protein

Histone protein H1 was modified *in vitro* using the highly reactive free radical (^•^OH). Structural changes in the protein due to OH radicals were investigated using biophysical studies. UV spectral observations suggested significant hyperchromicity (31.14%) in modified protein compared to native His-H1 (Table 2).

**TABLE 2:** Biophysical characterization of ROS-His-H1 protein.

Parameter	Native-His-H1	ROS-His-H1
Hyperchromicity (%) at 280 nm	—	+31.14%^*^
Intrinsic fluorescence (exc. At 275 nm)	—	+22.76%^*^
CD (secondary structures)		
α-helix	28 ± 2.2	22 ± 2.2 (−21.43%)^**^
β-sheet	33 ± 2.7	30 ± 2.7 (−9.09%)
Turns	21 ± 1.7	28 ± 1.7 (+33.33%)^**^
Random coil	18 ± 1.5	20 ± 1.5 (+11.11)

Signs–ve and +ve represent the increase and decrease compared to the unmodified protein. All test samples were run in triplicate. All values are calculated as mean ± standard deviation (SD). *p < 0.05 and **p < 0.01.

Furthermore, intrinsic fluorescence analysis showed similar findings, and there was a significant increase (+22.26%) in fluorescence in ROS-His-H1 compared to N-His-H1 (Table 2).

Secondary structural perturbations were also investigated in ROS-His-H1 by CD analysis. Remarkable changes were observed in all four components (α-helix, β-sheet, turns, and random coils) of the secondary structure due to OH radical modification. There was a significant decrease in the ‘α-helix’ structure (*p < 0.01*); however, a significant increase was observed in “turns” (*p < 0.01*) in ROS-His-H1 protein compared to N-His-H1 (Table 2).

### *In Vitro* Carbonyl Content Analysis

**FIGURE 1:**
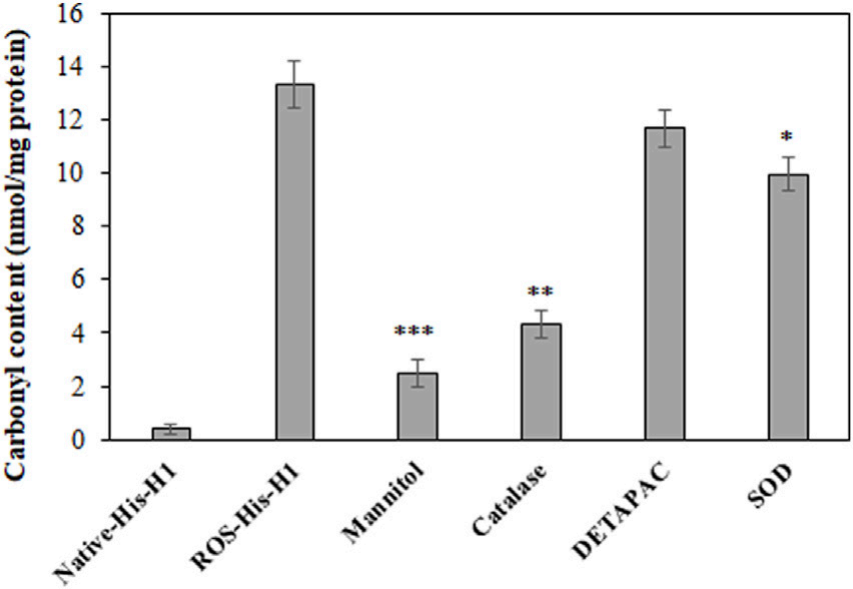
Detection of carbonyl compounds in native and ROS-modified histone H1 protein. Quenching studies using mannitol (100 mM), catalase (500 units/ml), DETAPAC (100 mM), and SOD (500 units/ml) were performed for the generation of carbonyl contents during protein oxidation. *p < 0.05, **p < 0.01, and ***p < 0.001.

The extent of *in vitro* protein oxidation can be observed using the levels of carbonyl compounds attached to protein molecules. ROS-modified protein was investigated for the levels of carbonyl contents and showed that a significant amount of the content (13.36 ± 0.9) was formed during OH radical oxidation reaction (Figure 1). To identify the role of free radicals in His-H1 protein modification, quenching studies were conducted. Different quenching agents (mannitol, catalase, DETAPAC, and SOD) were added to the reaction. Significant inhibitions in the formation of carbonyl content were recorded when mannitol (81.51%) was added to the reaction followed by catalase (67.59%) and SOD (25.29%) (Figure 1).

### *In Vivo* Oxidative Stress Analysis

**FIGURE 2:**
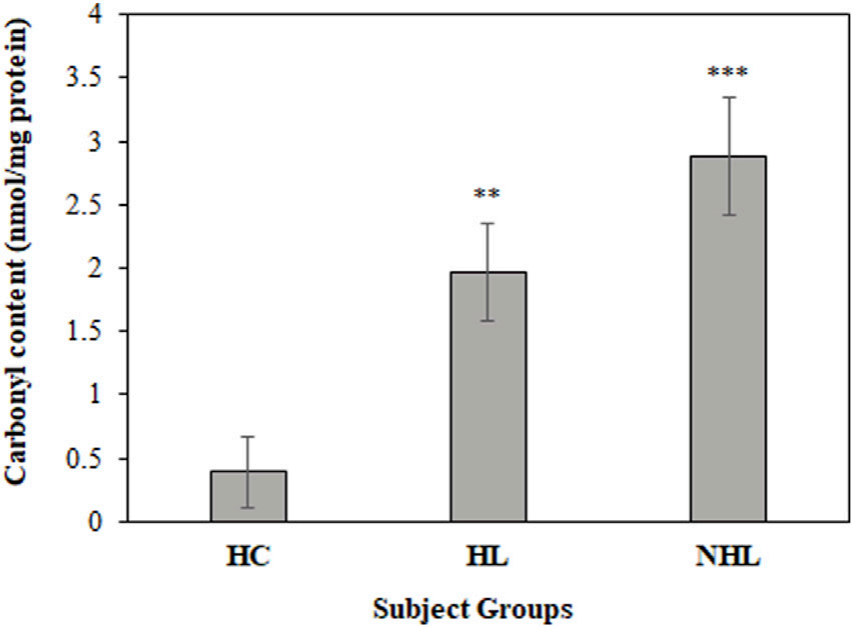
Detection of carbonyl contents in serum samples of HL and NHL patients. Healthy individuals were used as controls. **p < 0.01 and ***p < 0.001.

For a better insight on *in vivo* protein oxidation in different cancers, specifically for HL and NHL conditions, serum carbonyl compounds were detected in patients and healthy control samples. Both HL and NHL serum samples exhibited significant amount of carbonyl compounds (1.97 ± 0.38; *p* < 0.01 and 2.88 ± 0.47; *p* < 0.001, respectively) compared to healthy individuals (0.71 ± 0.28) (Figure 2).

**FIGURE 3:**
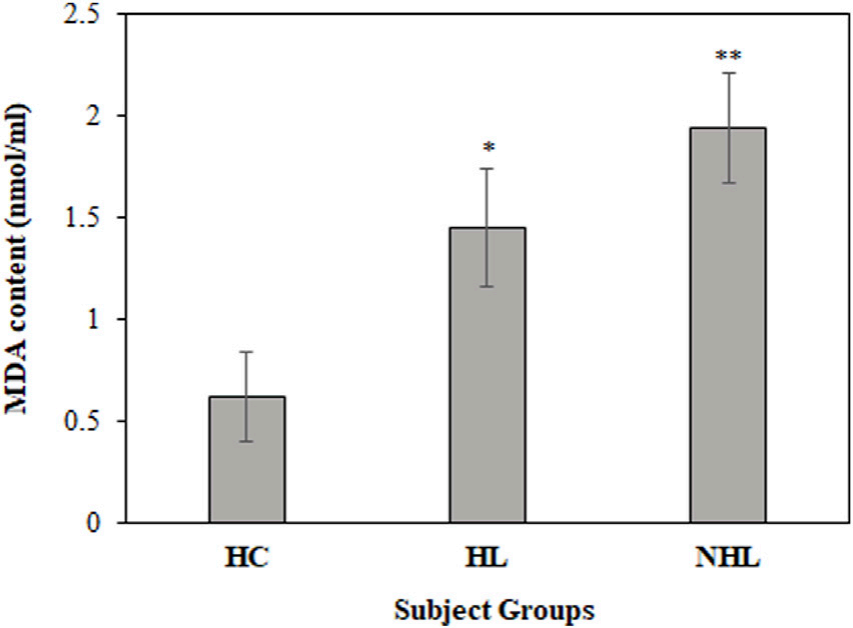
Detection of MDA content levels in serum samples of HL and NHL subjects. Serum samples from healthy individuals were used as controls. *p < 0.05 and **p < 0.01.

Furthermore, MDA levels are a hallmark for the estimation of *in vivo* oxidative stress during the disease state. Thus, all serum samples were analyzed to determine MDA levels. Serum samples from HL and NHL patients showed increased levels of the MDA content (1.45 ± 0.29; *p* < 0.01 and 1.94 ± 0.27; *p* < 0.001, respectively) compared to the healthy volunteers (0.62 ± 0.22) (Figure 3).

### Direct Binding ELISA

**FIGURE 4:**
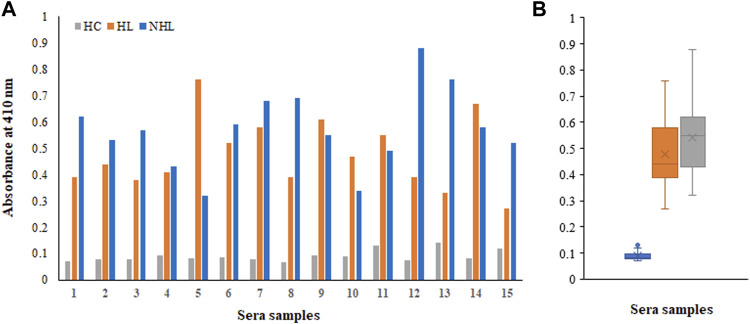
Detection of autoantibodies in serum samples from Hodgkin lymphoma (HL), non-Hodgkin lymphoma (NHL), and healthy control (HC) subjects against the ROS-His-H1 protein antigen using direct binding ELISA **(A)**. Range of absorbance for the autoantibody levels in all subjects **(B)**.

There were several biomarkers, which were investigated in lymphoma; however, no consensus was developed among any of them. We have used a new antigen ROS-modified His-H1 protein, and all the serum samples (lymphoma patients and healthy controls) were screened for the levels of autoantibodies against it. Direct binding ELISA results showed significantly high levels of serum autoantibodies in both HL (0.48 ± 0.14) and NHL (0.57 ± 0.16) cancer patients compared to healthy subjects (0.091 ± 0.022) (Figure 4A). However, the levels of autoantibodies in patients vary widely, ranging from 0.27 to 0.76 OD for Hodgkin lymphoma and from 0.32 to 0.88 OD for non-Hodgkin lymphoma subjects (Figure 4B).

**FIGURE 5:**
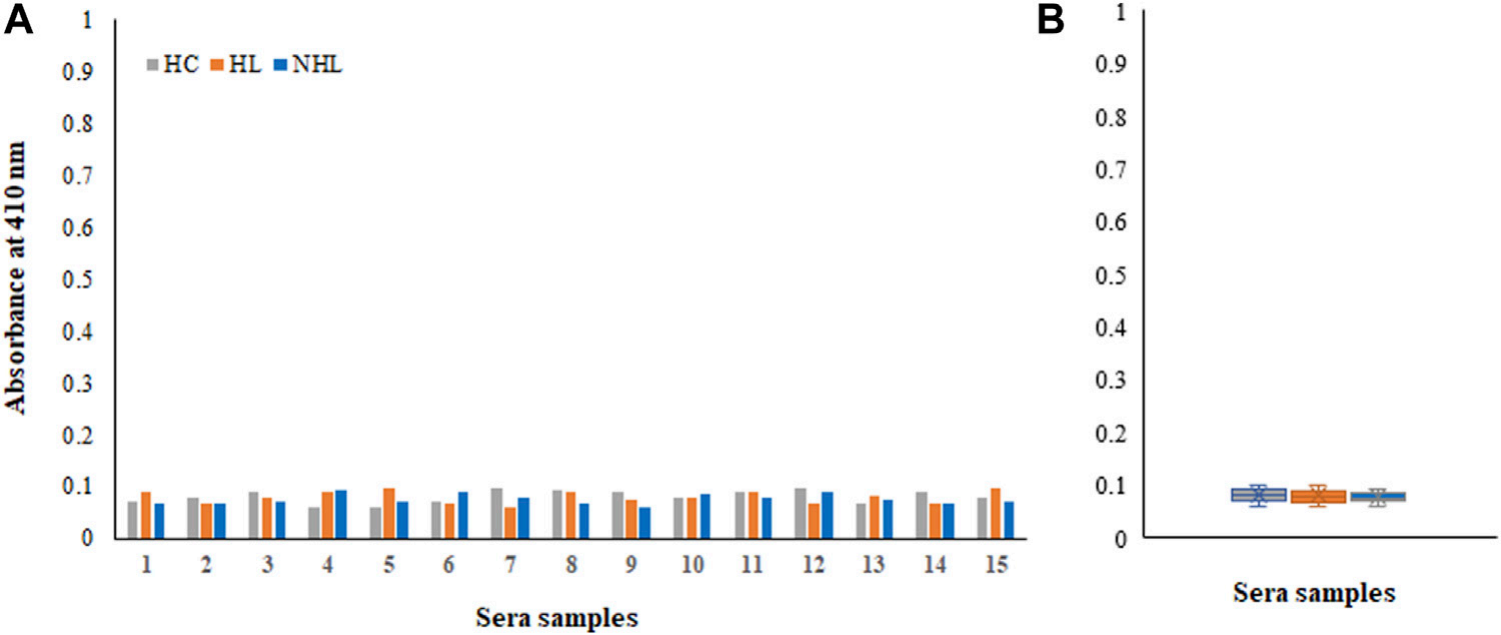
Detection of autoantibodies in serum samples from Hodgkin lymphoma (HL), non-Hodgkin lymphoma (NHL) and healthy control (HC) subjects against the non-modified His-H1 protein antigen using direct binding ELISA **(A)**. Range of absorbance for the autoantibody levels in all subjects **(B)**.

Moreover, samples from all cohorts exhibited negligible binding to the non-modified His-H1 protein (Figures 5A,B). Serum autoantibodies from patients and healthy subjects did not recognize the native form of the histone H1 protein.

### Inhibition ELISA

**FIGURE 6:**
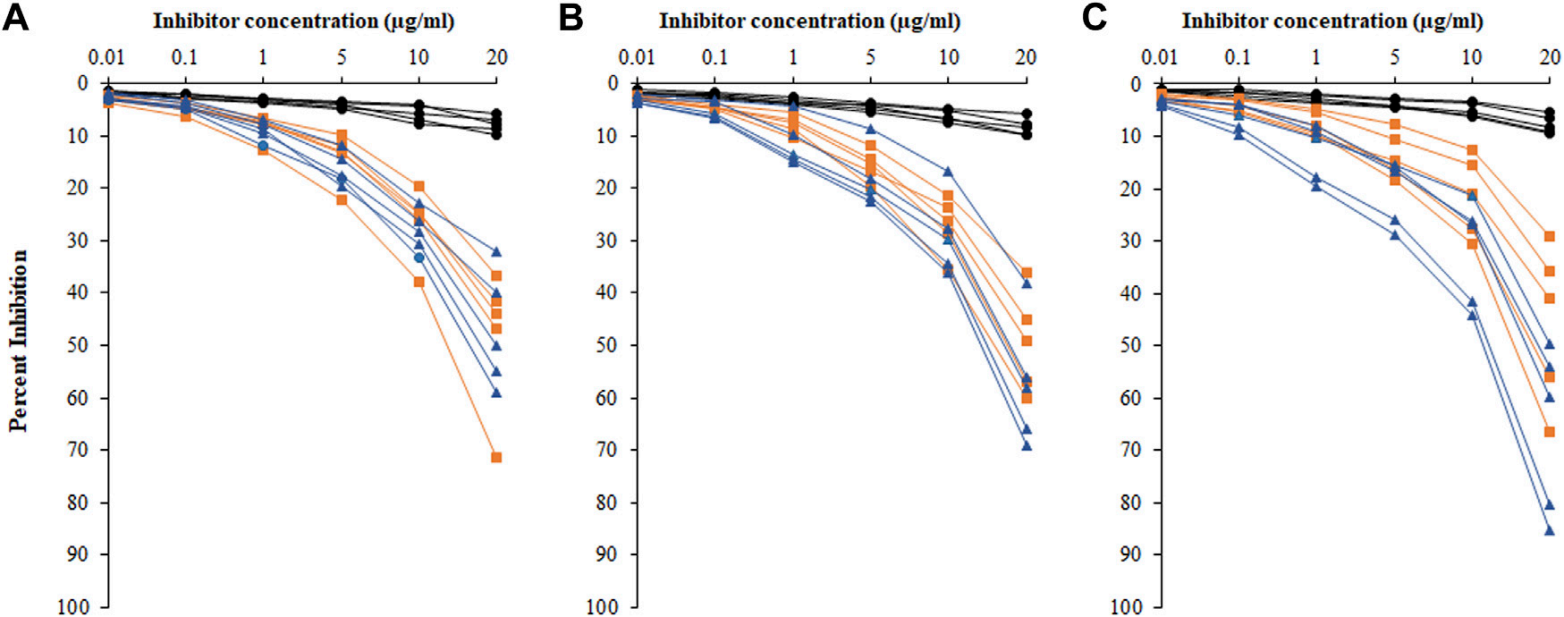
Percentage inhibition of serum autoantibodies was detected using ROS-His-H1 as an inhibitor with varying concentrations (0.01–20 μg/ml) for all samples from healthy control (-●-) HC, HL (-■-), and NHL (-▲-) subjects. Sample numbers 1–5 **(A)**; from 6 to 10 **(B)**; and from 11 to 15 **(C)** from all the cohorts. Serum samples of 1:100 dilutions were used in the assay. All samples were read on an ELISA reader at 410 nm. Each sample was run in triplicate.

Inhibition ELISA was used for the detection of serum autoantibodies and antigen specificities from HC, HL, and NHL subjects (Figure 6). Figures 6A–C represent competitive ELISA for five samples from each cohort. The specificity of the NHL serum autoantibodies exhibited mean maximum percentage inhibition at 20 μg/ml of 56.8 ± 14.56, with the ROS-His-H1 antigen followed by HL cancer patients of 47.7 ± 12.19. However, very low mean maximum percentage inhibition (7.92 ± 1.59) was observed in HC subjects.

### Quantitation of Immune Complex by the Langmuir Plot

**FIGURE 7:**
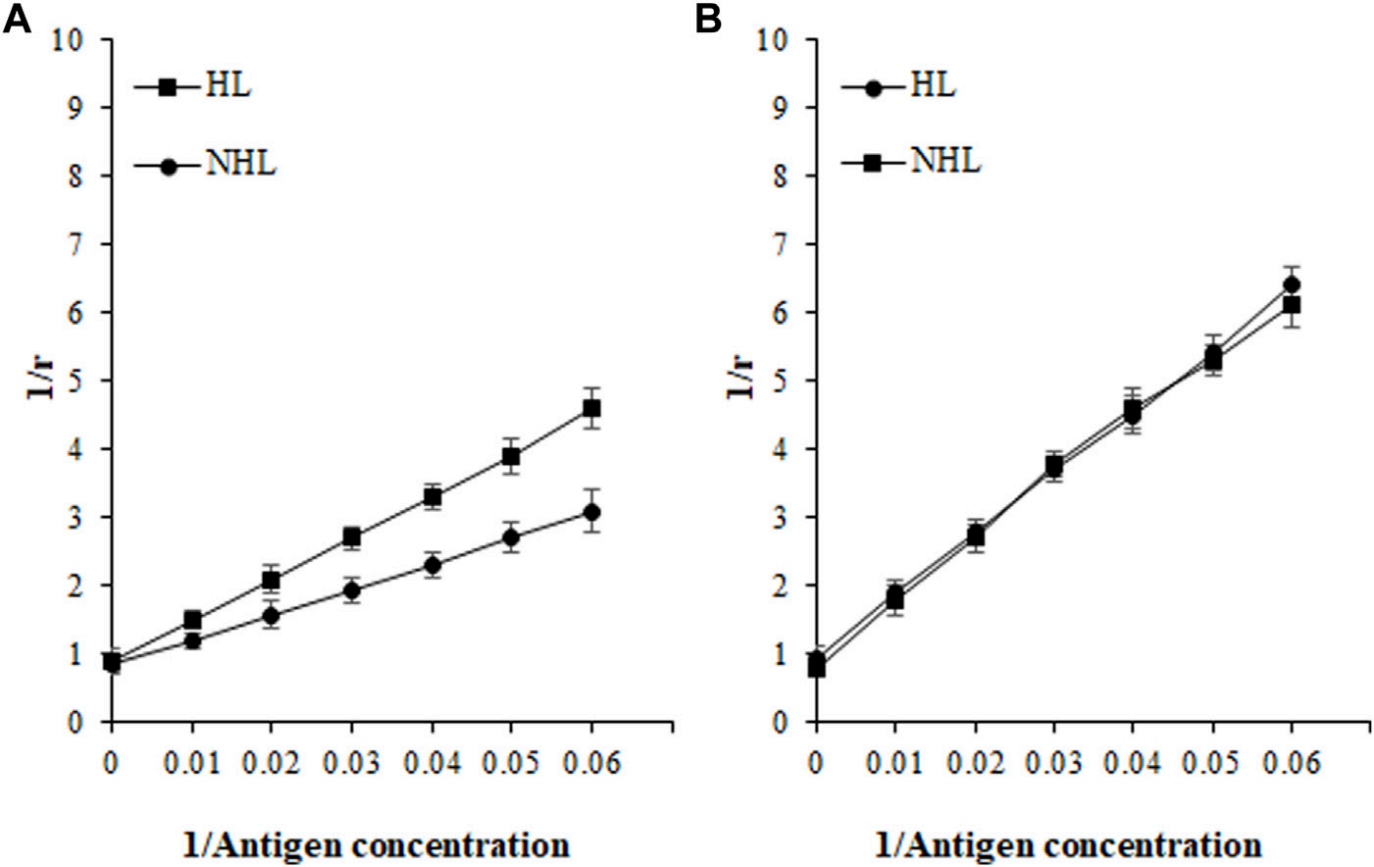
Apparent association constants were calculated using the Langmuir plot for antigens ROS-His-H1 **(A)** and His-H1 **(B)**. A constant amount of IgG (100 µg) was incubated with different concentrations of antigen (0–40 µg) for 2 h at room temperature and overnight at 4°C. Langmuir plot of the reciprocal of the bound antigen concentration to antibody (1/r) versus the reciprocal of the free antigen concentration. ROS-His-H1 antigen and IgG binding for HL (-■-) and for NHL subjects (-●-).

Based on affinity chromatography, IgG from serum samples of HL (*n* = 5; sera number 5, 7, 9, 11, and 14) and NHL (*n* = 5; 1, 7, 8, 12, and 13) subjects were isolated using the protein A agarose column. Five samples from each cohort were selected based on the highest absorbance in direct binding ELISA. Quantitative precipitation titration curves were used to identify antigen-antibody affinity. In this assay, an increasing amount of antigens (ROS-His-H1 and His-H1) were incubated with a constant amount of isolated IgG (100 µg). The mean maximum amount of 26.3 ± 1.3 µg of ROS-His-H1 antigen was bound to 52.8 ± 1.1 µg of IgG from HL subjects (*n* = 5). Similarly, the mean maximum amount of 21.1 ± 0.9 µg of ROS-His-H1 was bound to 66.7 ± 1.2 µg of IgG from NHL subjects (*n* = 5). ACCs were evaluated using the Langmuir plot. ACCs of IgG from HL and NHL subjects were computed to be 1.33 × 10^–6^ M and 1.46 × 10^–6^ M, respectively, against ROS-His-H1 (Figure 7A). However, comparatively fewer ACCs were computed for non-modified His-H1 protein for HL (2.38 × 10^–8^ M) and NHL (2.46 × 10^–8^ M) subjects (Figure 7B).

## Discussion

Post-translational modifications in the histone protein led to chromatin remodeling, which in turn resulted in sustained pro-inflammatory gene transcription (Ghizzoni et al., 2011; Cross et al., 2019). The sustained and uncontrolled inflammatory cytokine production causes inflammation (Hirano, 2021). At the site of inflammation, reactive oxygen and reactive nitrogen species are produced by these inflammatory cytokines (Reuter et al., 2010). The production of free radicals can cause DNA damage and promote DNA mutations (Klaunig et al., 2010). Cancer cells mainly generate energy through anaerobic glycolysis, leading to high levels of ROS and reactive di-carbonyl compounds such as methylglyoxal (MGO) (Lee and Chang, 2014; Volpe et al., 2018). Previous publications based on the hydroxyl radical modification of proteins, which exert extensive damage to the proteins, were analyzed by spectroscopic and fluorescence studies (Rasheed et al., 2006; Ali Khan et al., 2007; Ahmed et al., 2009). In coherence to these studies, the ROS-modified histone H1 protein exhibited hyperchromicity and an increase in the fluorescence intensity which might be due to structural perturbations in our study. Furthermore, significant alterations were also observed in the secondary structures after ROS damage to the ROS-His-H1 protein.

In several chronic diseases such as cancer, diabetes, and atherosclerosis, the generation of ROS and its homeostasis might get out of control, causing severe oxidative stress concomitantly with decreased levels of antioxidant compounds (Karihtala et al., 2006; Matés et al., 2008; Haidari et al., 2010). Similar conditions were also observed in lymphomas (Peroja et al., 2012). In our study, HL and NHL patients’ serum samples exhibited an increased amount of protein oxidative marker “carbonyl compounds” compared to the healthy individuals. Furthermore, another well-known oxidative marker MDA was found to be significantly higher in both HL and NHL cancer serum samples as compared to the healthy individuals.

Post-translational modifications in histone proteins predispose cancer initiation and progression at the site of inflammation (Rajendrasozhan et al., 2009). Hence, increased serum levels of pro-inflammatory cytokines TNF-α and IL-6 were found in both HL and NHL patients; however, another pro-inflammatory cytokine IFN-γ did not show significant changes compared to the healthy individuals.

It is a well-known fact that there is the presence of circulatory autoantibodies against nuclear materials in cancer patients known as anti-nuclear antibodies (Feist et al., 2007). Due to these reports, we designed this study to evaluate the levels of autoantibodies in both lymphoma patients against ROS-modified histone H1 protein. Direct binding ELISA findings exhibited significantly high levels of circulatory autoantibodies detected in HL and NHL patients, which was further ascertained by the more specific assay “inhibition ELISA”.

Oxidative stress and chronic inflammation during lymphoma might cause immune imbalance *via* the generation of endogenous antigens (Kidane et al., 2014). After a cascade of reactions, these autoantigens might generate autoantibodies which can interact with each other and form immune complexes (Khan and Ali Khan, 2015; Alouffi et al., 2018). The subtle reactions are crucial in determining the disease state. In the current study, the formation of IgG from patients and ROS-modified protein antigen complexes were determined using the Langmuir plot. The highest AAC was calculated for ROS-His-H1 against NHL patients’ IgG compared to HL subjects’ IgG, whereas the non-modified protein His-H1 showed remarkably low ACC for IgG from HL and NHL patients. This might correlate with the increased levels of inflammatory cytokines and oxidative stress markers in NHL compared to HL patients.

Thus, during HL and NHL, there is imbalance homeostasis of oxidative stress concomitantly with inflammatory conditions as there might be the possibilityof modifications in histone H1 protein, which induced the protein to exhibit its cryptic epitopes. These epitopes may be identified as an antigen by our immune system, resulting in the production of autoantibodies. The oxidative stress-induced chromatin structure together with autoantibody production further tilts the balance of the immune system in lymphoma patients, leading to the possible epigenetic dysregulations.

## Conclusion

Hodgkin’s lymphoma and non-Hodgkin’s lymphoma are common cancers. Cancers initiate oxidative stress, which imbalances antioxidant redox state-regulating enzymes, leading to DNA damage. Histone is a protein that is present with the DNA and plays an important role in stabilizing the chromatin structure. In this study, ROS modification of histone H1 induced structural perturbation generating/exposing neo-epitopes against which autoantibodies were generated in both HL and NHL patients. Antigen-antibody binding was evaluated using ELISA, and their specificity was ascertained by inhibition ELISA. ACC showed higher recognition for IgG from NHL and ascertained more immune imbalance, which was supported by increased levels of inflammatory cytokines and oxidative markers (MDA and carbonyl compounds). Thus, histone H1 modifications might affect the histone DNA dynamics and are correlated with gene dysregulation. These subtle molecular changes might further aggravate the disease.

## Data Availability

The original contributions presented in the study are included in the article, further inquiries can be directed to the corresponding author.
